# Supplementary figures and images for: The activity of human enhancers is modulated by the splicing of their associated lncRNAs
Source: PLoS Comput Biol. 2022 Jan 11;18(1):e1009722. doi: 10.1371/journal.pcbi.1009722 (PMC8803168; doi:10.1371/journal.pcbi.1009722)

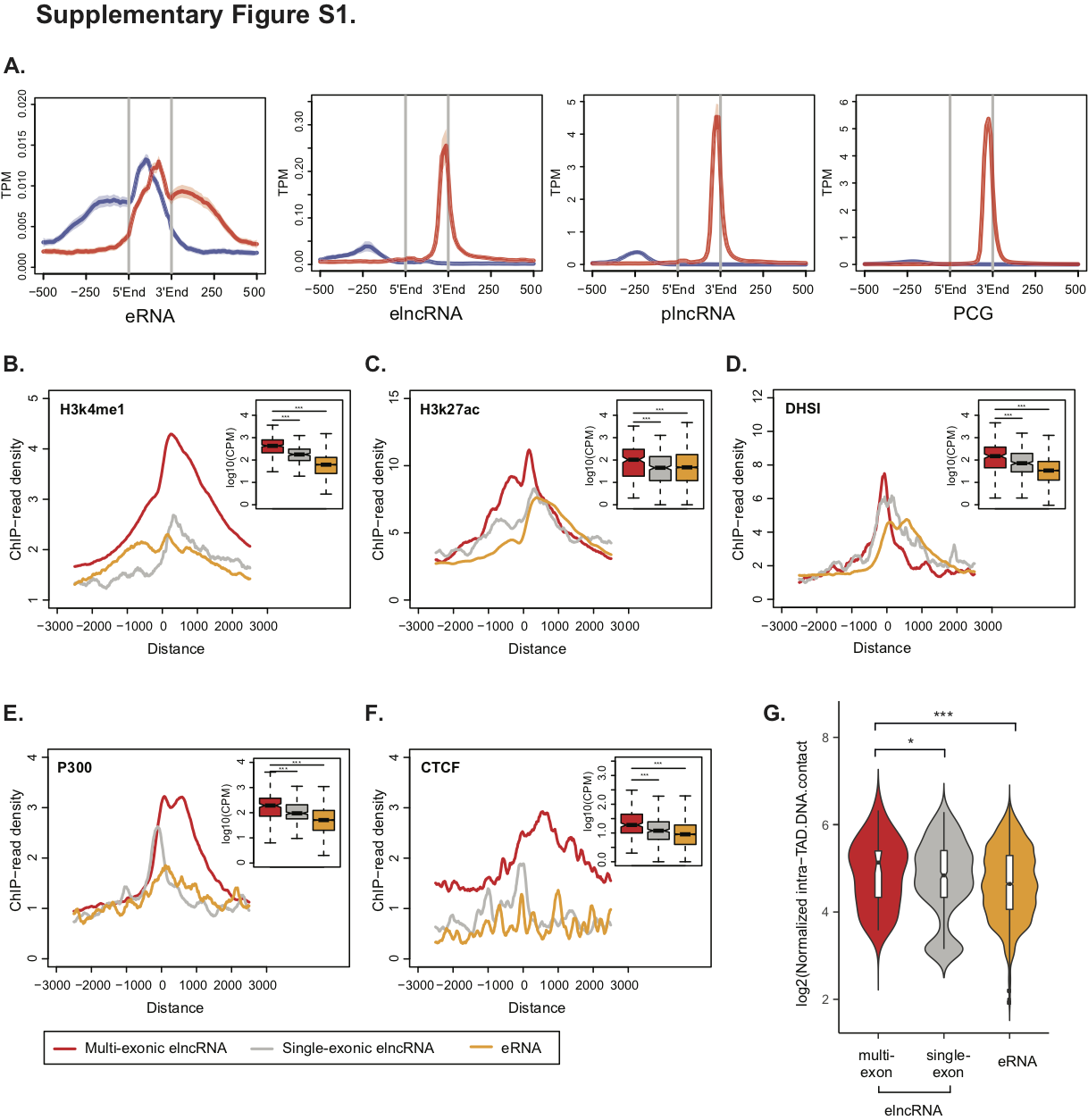

Supplement: S1 Fig — (A) Metagene plots of CAGE reads centered at transcription initiation regions (TIRs) of eRNAs, and promoters (estimated as -500bp to annotated gene TSS) of elncRNAs, plncRNAs and protein-coding genes (PCGs). Sense (red) and antisense (blue) reads denote those that map to the same or opposite strand, respectively, as the direction of their cognate TIRs. Metagene plots and distribution (figure insets) of (B) H3K4me1, (C) H3K27ac, (D) DNase I hypersensitive sites (DHSI), (E) P300 and (F) CTCF ChIP-seq reads in LCLs at promoters of multi-exonic (red) and single-exonic (grey) elncRNAs, and eRNAs (yellow). (G) Distribution of the average amount of DNA:DNA contacts within LCL TADs containing multi-exonic (red) and single-exonic (grey) elncRNAs and eRNAs (yellow). Differences between groups were tested using a two-tailed Mann-Whitney U test. * p < 0.05; *** p < 0.001. (TIFF) [file pcbi.1009722.s001.tiff]

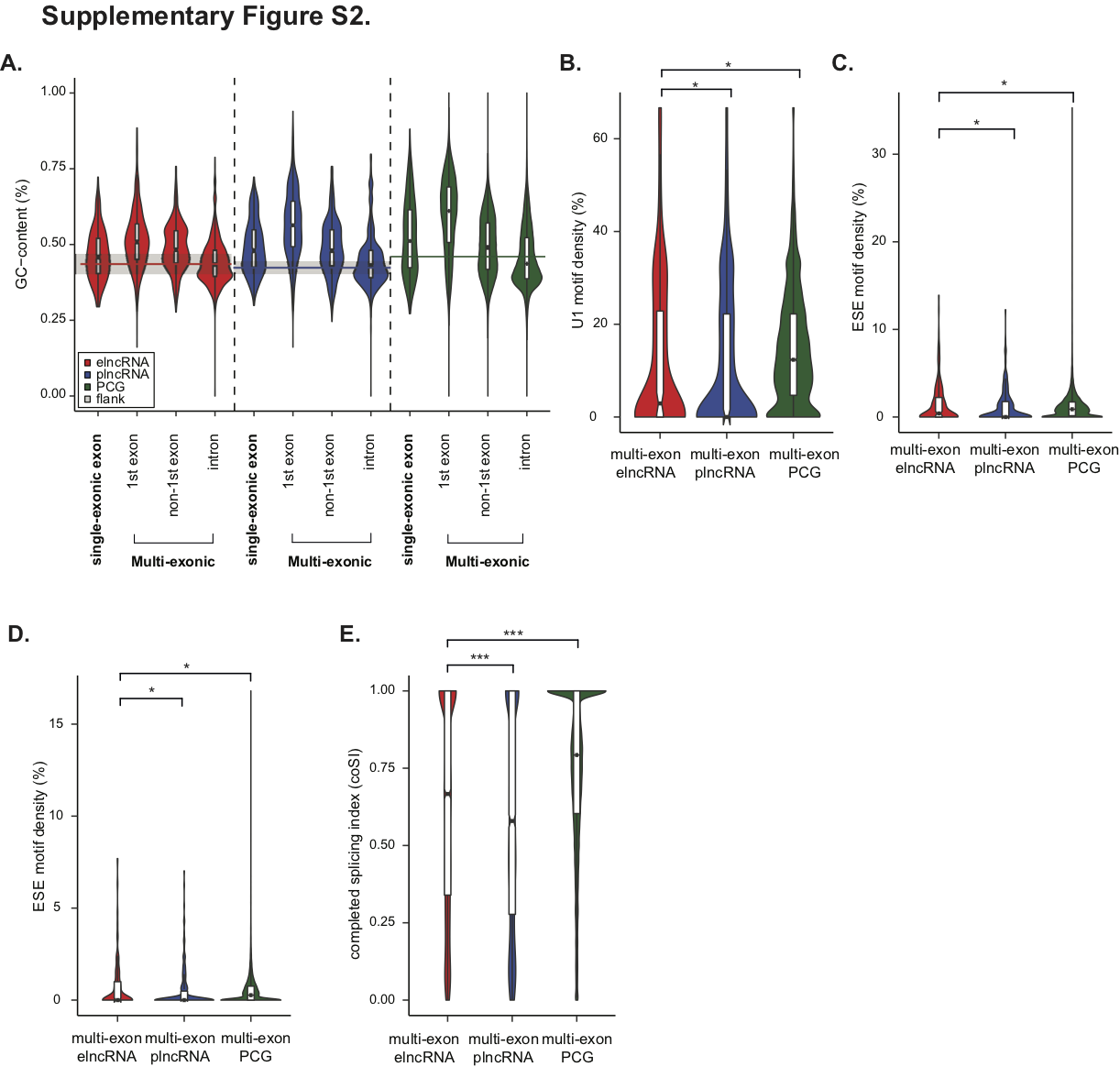

Supplement: S2 Fig — (A) Distribution of GC-content across exons and introns of elncRNAs (red), plncRNAs (blue), and protein-coding genes (green). Distribution of the density of predicted (B) U1 spliceosome RNAs (snRNPs) and exonic splicing enhancers (ESEs), using (C) a comprehensive (n = 238) and (D) a smaller but more stringent set (n = 54) of predicted ESE motifs, within multi-exonic elncRNAs (red), plncRNAs (blue) and protein-coding genes (green). (E) Distribution of the completed splicing index (coSI) for multi-exonic elncRNAs (red), plncRNAs (blue) and protein-coding genes (green). Differences between groups were tested using a two-tailed Mann-Whitney U test. * p < 0.05; ** p < 0.01; *** p < 0.001; NS p > 0.05. (TIFF) [file pcbi.1009722.s002.tiff]

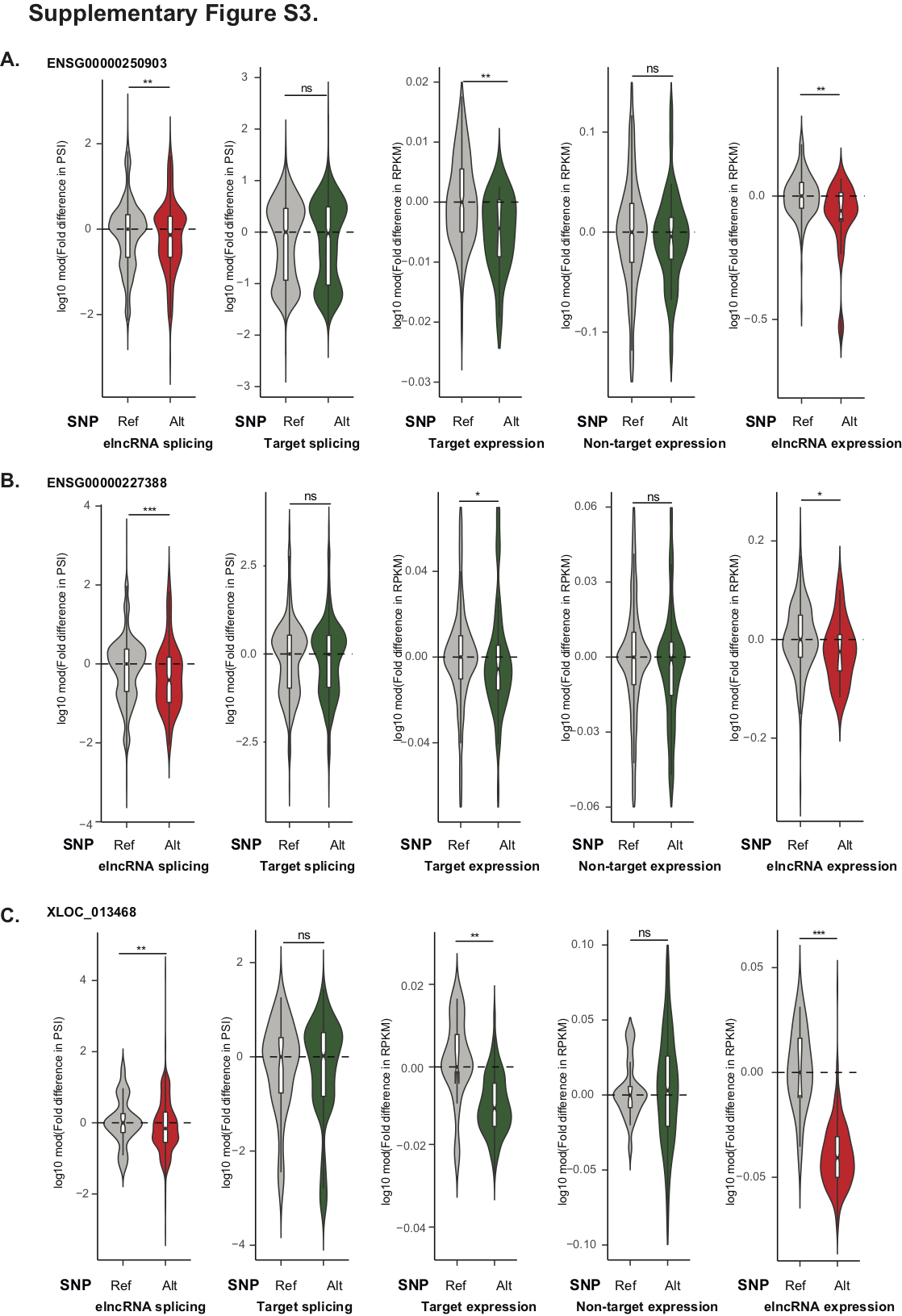

Supplement: S3 Fig — Distribution of the log10 modulus fold difference in splicing (PSI), relative to the median of samples with reference genotype, between individuals that carry alternative or reference alleles at elncRNA splice site of (A) ENSG00000250903, (B) ENSG00000227388 and (C) XLOC_013468 of all directly affected splicing events of the elncRNA and all splicing events of their target protein coding genes (1, 7, and 1 targets, respectively); as well as fold difference in expression levels (RPKM) of targets, non-targets and the elncRNA. Differences between groups were tested using a two-tailed Mann-Whitney U test. * p < 0.05; ** p < 0.01; *** p < 0.001; NS p > 0.05. (TIFF) [file pcbi.1009722.s003.tiff]

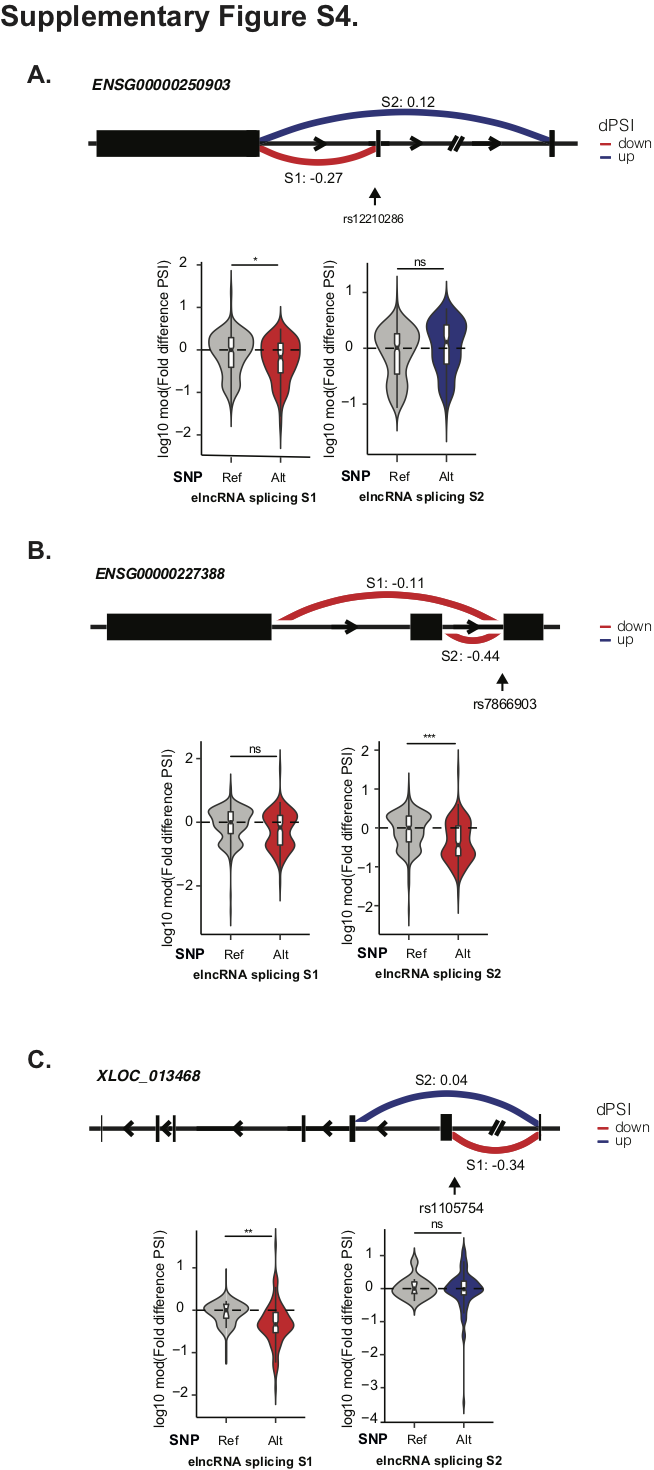

Supplement: S4 Fig — (Top panels) Representation of the differential splicing events between samples with different genotypes for elncRNAs, (A) ENSG00000250903, (B) ENSG00000227388 and (C) XLOC_013468. Median differential splicing (log10 modulus fold difference in Percentage-Spliced-In (dPSI)) of each splicing event is noted next to the arrow. Decreases are represented in red and increases represented in dark blue. (Bottom panels) Distribution of the log10 modulus fold difference in PSI, relative to the median of samples with reference genotype, between individuals that carry alternative or reference alleles at each corresponding elncRNA splicing event as shown in the top panels. Differences between groups were tested using a two-tailed Mann-Whitney U test. * p < 0.05; ** p < 0.01; *** p < 0.001; NS p > 0.05. (TIFF) [file pcbi.1009722.s004.tiff]

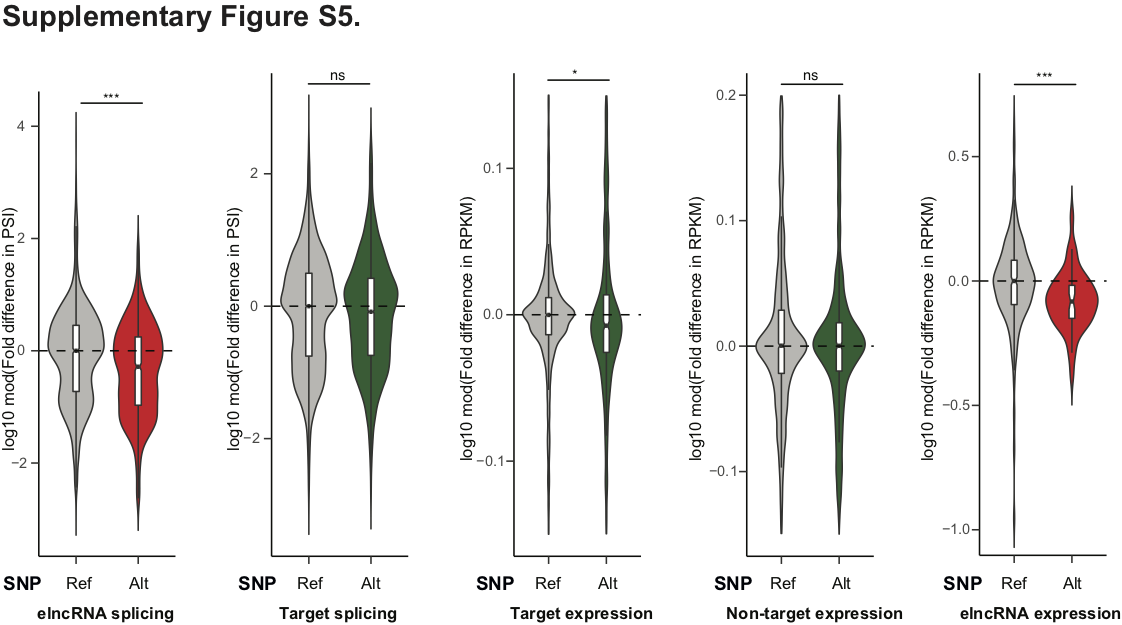

Supplement: S5 Fig — Distribution of the median log10 modulus fold difference, relative to the median of samples with reference genotype, between individuals of the Yorubin (YRI) population that carry alternative or reference alleles at elncRNA splice sites, in splicing (PSI) of multi-exonic elncRNAs and target protein coding genes; target and non-target gene expression levels (RPKM); and elncRNA expression. Differences between groups were tested using a two-tailed Mann-Whitney U test. * p < 0.05; ** p < 0.01; *** p < 0.001; NS p > 0.05. (TIFF) [file pcbi.1009722.s005.tiff]

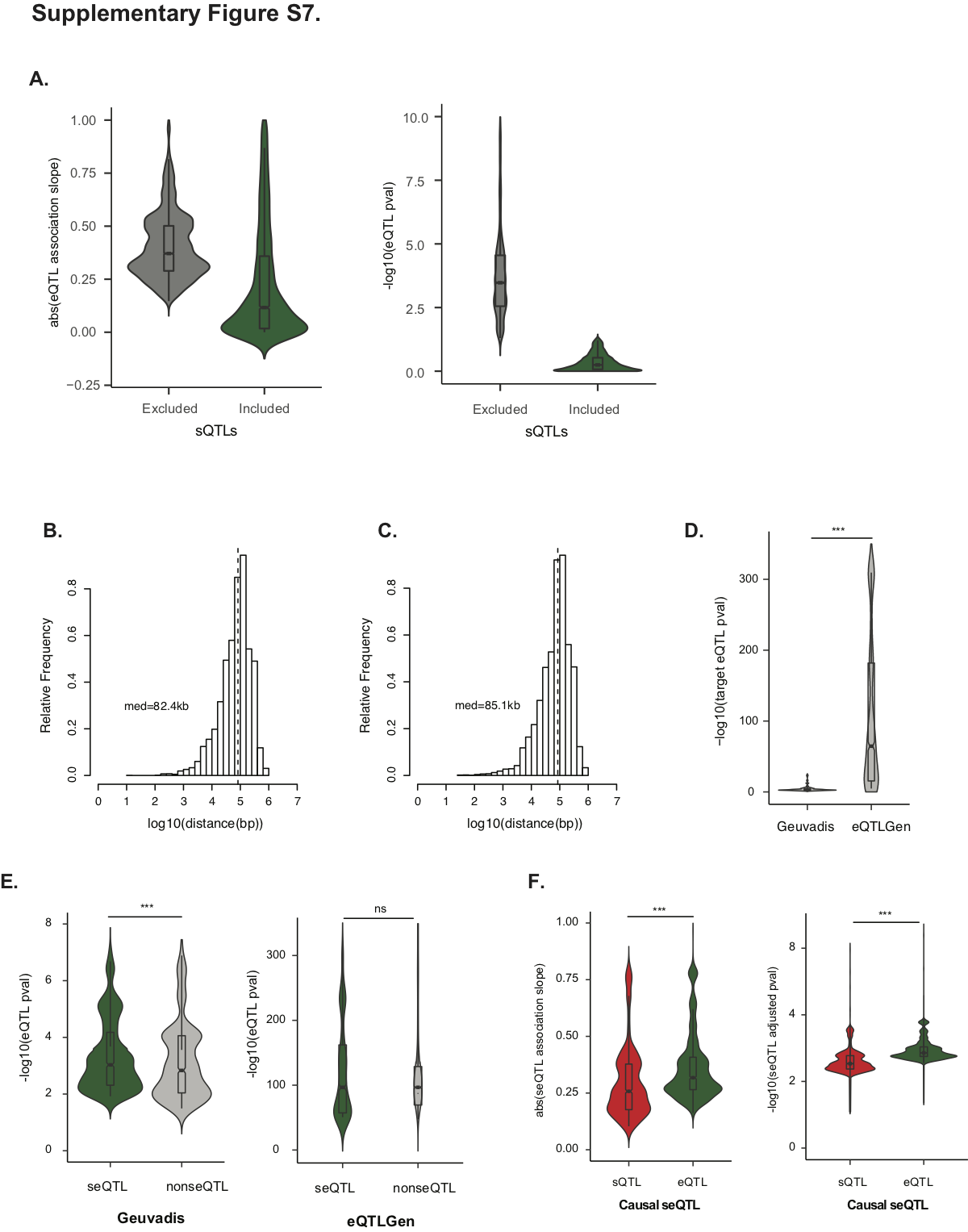

Supplement: S7 Fig — (A) Distribution of the absolute slope (left panel) and the -log10 of the adjusted p-value (right panel) of elncRNA sQTLs that are excluded (grey) or included (green) in analysis due to their significant associations with elncRNA expression, respectively. Distribution of distance between genomic coordinate of seQTL and position of their associated elncRNA annotated transcriptional start sites (B) and enhancers (C). (D) Distribution of elncRNA sQTLs that are also associated with a cis-target expression (seQTL) as identified using a LCL population with 373 samples (Geuvadis) or blood samples (eQTLGen) with 31,684 samples. (E) Distribution of elncRNA seQTLs and non-seQTLs (elncRNA sQTLs not associated with target expression in LCL population) associations as identified in LCL Geuvadis population or in blood eQTLGen samples. (F) Distribution of the absolute slope (left panel) and the -log10 of the adjusted p-value (right panel) of elncRNA sQTL association (red) and target eQTL association (green) for all causal seQTLs. Differences between groups were tested using a two-tailed Mann-Whitney U test. * p < 0.05; ** p < 0.01; *** p < 0.001; NS p > 0.05. (TIFF) [file pcbi.1009722.s007.tiff]

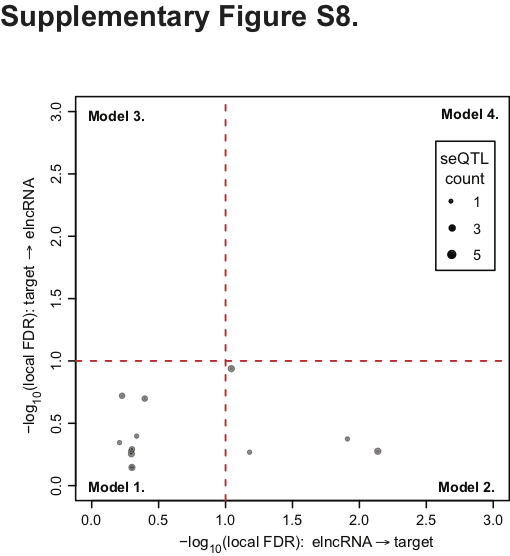

Supplement: S8 Fig — Scatterplot depicting the best joint elncRNA seQTLs (the most significant variant associated with elncRNA splicing) used to infer relationship between elncRNA splicing and cis-target expression levels using causal inference testing, as illustrated using local FDR associated with the four models (as illustrated in Fig 4B). Dotted black lines denote significance threshold at local FDR < 0.1. (TIFF) [file pcbi.1009722.s008.tiff]

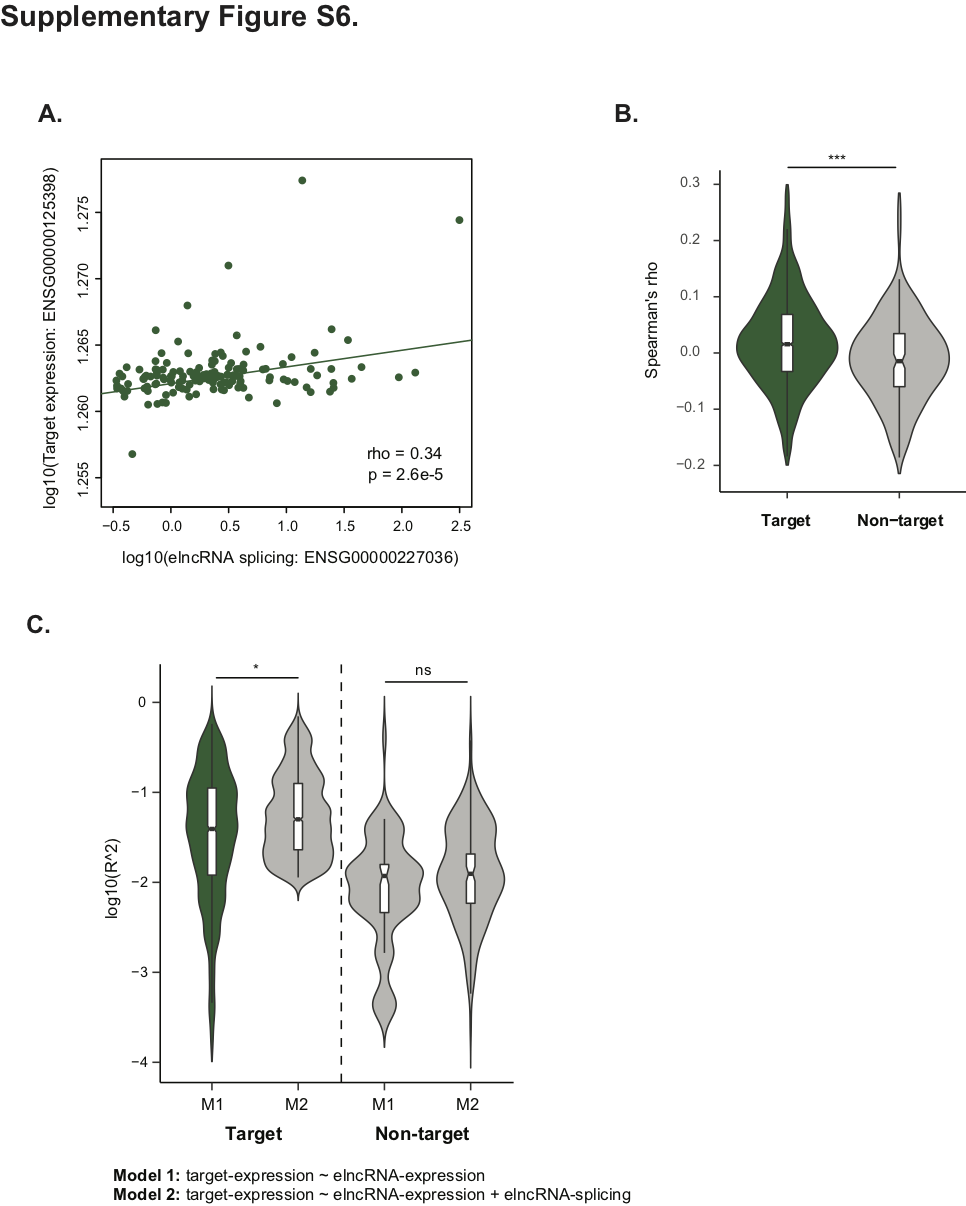

Supplement: S9 Fig — Distribution of the absolute slope or z-score (left panels) and adjusted p-value (right panels) of elncRNA target eQTL associations that can be replicated (after multiple-testing correction <5% and in the same association direction, green) or not (grey) using LCL samples from GTEx (A) and blood samples from eQTLgen (B). (TIFF) [file pcbi.1009722.s009.tiff]
