# Supplementary material for: The activity of human enhancers is modulated by the splicing of their associated lncRNAs
Source: PLoS Comput Biol. 2022 Jan 11;18(1):e1009722. doi: 10.1371/journal.pcbi.1009722 (PMC8803168; doi:10.1371/journal.pcbi.1009722)
Supplement: Attachment — Submitted filename: elncRNA_RC_response_to_reviewers.pdf [file pcbi.1009722.s013.pdf]

We would like to thank the reviewers for their constructive feedback. We respond to all the reviewers points below. We highlighted major changes introduced to the manuscript in response to both reviewers' comments in the attached revised version of the manuscript.

Reviewer #1 (Evidence, reproducibility and clarity (Required)):

The work described in this manuscript by Tan and Marques aims to address if the splicing of enhancer-associated long noncoding RNAs (elncRNAs) has a direct impact on enhancer activity or just reflects their cognate's enhancer's high activity.

For this purpose, the authors started by integrating RNA-seq data for human lymphoblastoid cell lines with ENCODE enhancer annotations and ChIP-seq data for enhancer function-associated chromatin modifications to show that multi-exonic elncRNAs are more transcriptionally active than single-exonic elncRNAs and eRNAs. They then show that regions flanking elncRNA splice sites are enriched in splicing-associated sequence elements and that these are under stronger purifying selection (suggesting some functional relevance), both when compared to promoter-associated lncRNAs. They also show the concomitance of cis-disrupted splicing in elncRNAs and drops in expression in their target genes. Finally, they use causal inference analysis of joint seQTLs and joint scQTLs to investigate the causal relationship between splicing of elncRNAs and expression of putative gene targets and chromatin states at elncRNA cognate enhancers, respectively. They conclude that, in both cases, most associations are causally mediated by splicing of elncRNAs and therefore that this contributes to their enhancer activity.

This manuscript is generally well written, targets an original question and potentially sets the seeds for a new exciting line of research on transcriptional regulation, by providing some evidence for the functional relevance of the splicing of elncRNAs. However, the overlooking of some important aspects of the regulation of RNA splicing led to biases in the design of data analyses and in the interpretation of the biological implications of some results that need to be dealt with before the described work can be considered sound enough for publication.

We would like to thank the reviewer for taking the time to assess our manuscript and for the constructive comments.

Essential revisions:

1.Results, page 10, lines 21-24: The statement that "the impact of SS variants on gene splicing efficiency depends on the total number of alternative transcripts and exons" is not properly substantiated. The four examples given in Figure S3 do not illustrate any dependence or trend. If such dependence is "expected" the underlying concept must be explained, i.e. why the impact of SS variants on splicing efficiency should depend on the number of alternative transcripts and exons. The reader is unrealistically expected to be used to the chosen splicing efficiency metric to intuit its dependence on the number of exons. Moreover, it is not obvious where the dependence on the number of alternative transcripts comes from, particularly given that alternative splicing (e.g. the skipping of a neighbouring exon, if internal) is not profiled.

In the previous version of the manuscript, we estimated gene level changes in splicing, which includes all alternative splicing events within a gene. Therefore, the more exons an elncRNA has, the more “diluted” we expect the overall impact of SS variant on elncRNA splicing efficiency to be. After considering the reviewer’s comment, we realized that only splicing events directly impacted by the SS variant should be considered in this analysis.

In the revised version of the manuscript, we considered only alternative splicing events that include the splice donor acceptor site changed by the SS variant, and are therefore a direct consequence, of the SS variant. As suggested by the reviewer, for these splicing events, we report the fold difference in Percentage-Spliced-In (PSI) (estimated by Leafcutter (Li et al. 2018)) between samples that carry reference and alternative alleles at these SS variants. To further illustrate these changes, we now include a diagram, for each SS variant, with differential splicing information and the fold difference in PSI for each affected splicing events (Figure 3B,C, Supplementary Figure S3). In addition, the overall change across all affected splicing events is also plotted in Figure 3D and Supplementary Figure S4.

We have modified this section to account for this and the next comment from the reviewer.
“*To estimate the impact of SS variant on splicing efficiency, we calculated the Percentage-Spliced-In (PSI) (Li et al. 2018) per individual and for each elncRNA splicing event involving the splice donor or acceptor site disrupted by the SS variants (Figure 3B,C, Supplementary Figure S3). PSI measures exon inclusion and considers spliced reads spanning exon junctions (Li et al. 2018). We compared the average difference in PSI, as a proxy for change in splicing efficiency, of all affected splicing events between individuals that carry the reference and alternative canonical splice donor/acceptor sites (GT-AG). Alongside decreased exon inclusion, SS variants can also promote exon skipping events (Figure 3B,C, Figure S3). Despite some increase in exon skipping, SS variants are associated with an overall decrease in splicing efficiency (Figure 3D and Supplementary Figure S4).*” (Page 10).

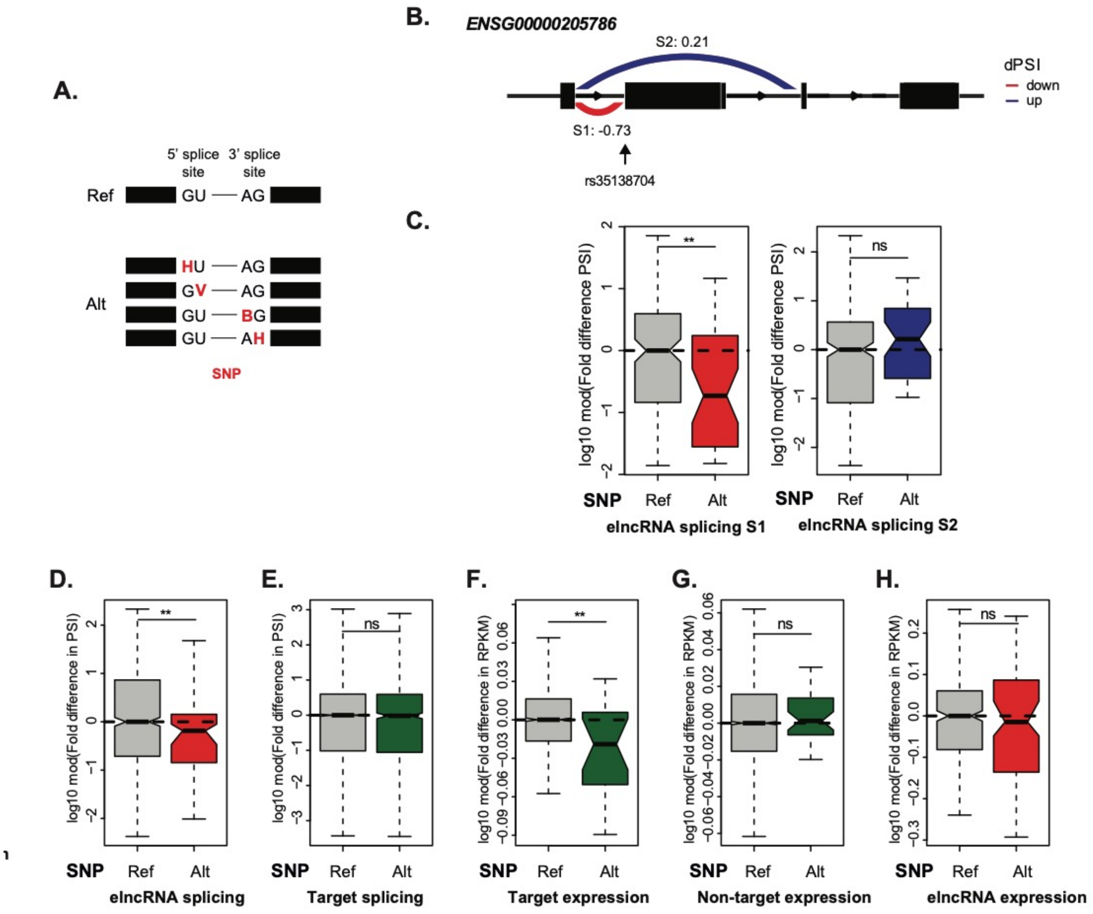

Revised Figure 3. Disrupted elncRNA splicing impacts *cis*-gene regulation. (A) Examples of variants that can disrupt elncRNA splicing. In contrast to individuals that carry the reference genome allele at a canonical splice site, those with alternative alleles have at least one variant that disrupt the GU dinucleotide at splice donor site (denoted as HU or GV) or AG dinucleotide at splice acceptor site (denoted as BG or AH). (B) Representation of the differential splicing events between samples with different genotypes for one elncRNA, ENSG00000205786. Median differential splicing (log10 modulus fold difference in Percentage-Spliced-In (dPSI)) of each splicing event is noted next to the arrow. Decreases are represented in red and increases represented in dark blue. (C) Distribution of the log10 modulus fold difference in PSI, relative to the median of samples with reference genotype, between individuals that carry alternative or reference alleles at each corresponding elncRNA splicing event as shown in (B). (D-H) Distribution of the log10 modulus fold difference in splicing (PSI), relative to the median of samples with reference genotype, between individuals that carry alternative or reference alleles at elncRNA splice site of ENSG00000205786, of all directly affected splicing events of the (D) elncRNA and (E) all splicing events of its target protein coding genes (5 targets); as well as fold difference in expression levels (RPKM) of (F) targets, (G) non-targets and (H) the elncRNA. Differences between groups were tested using a two-tailed Mann-Whitney *U* test. * $p < 0.05$; ** $p < 0.01$; *** $p < 0.001$; NS $p > 0.05$.

2. Along these same lines, and more importantly, why haven't the authors looked at the possibility that a variant disrupting a splice site would lead to skipping of the neighbouring exon (if internal)? Given how the spliceosome operates (in terms of intron and exon recognition), wouldn't this be the most likely scenario? When calculating the splicing index, are reads spanning junctions between non-consecutive exons considered? Otherwise, not profiling alternative isoforms generated by exon skipping will necessarily bias splicing efficiency quantifications by overlooking fully efficient splicing associated with such isoforms. Similarly, how did the authors make sure that splicing changes did not bias elncRNA expression estimates? How was the effective transcript length determined for the calculation of RPKMs? The authors need to make these methodological clarifications, as well as why exon skipping was not considered as a splicing disruption with potential functional implications. Calculating the percent spliced-in (PSI) for all internal exons would be much informative.

Regarding the methodology, what we refer to as splicing efficiency is Percentage-Spliced-In (PSI). We calculated PSI for all, including alternative, splicing events. We now make this clearer throughout the manuscript and in the figure axis/legends.
As detailed in the methods section, to minimize the impact of alternative splicing on gene expression estimates, we quantified expression at the gene, and not at the transcript, level using HTSeq across all annotated exons. This approach allows us to assess elncRNA and target gene expression while masking differences in alternative transcript abundance, which are not relevant in the context of this analysis.

As suggested by the reviewer, instead of considering PSI of all possible splicing events of the gene, in the revised version of the manuscript, we considered only splicing events that are directly impacted by the SS variant. This change does not impact our conclusions, but certainly provides a better understanding of how SS variants impact splicing and we would like to thank the reviewer for raising this point. As predicted by the reviewer and as expected given how the spliceosome operates, exon skipping is a frequent outcome of SS variants. However, the increase in exon skipping is not sufficient to compensate for the decrease in the inclusion of these exons, which is directly impacted by the SS variants. This is demonstrated by the lower overall splicing efficiency for each elncRNAs in individuals that carry SS variants that disrupt canonical splice/donor acceptor sites (Figure 3D and Supplementary Figure S4).

3.All results in panels 3B-F are presented as fold differences. It is actually not clear what those differences refer to. For instance, the grey boxes are the distributions of the fold differences in splicing index / expression between individuals carrying reference alleles and what?

The boxplots represent the distribution of the fold difference in PSI or expression for each individual relative to the median PSI or expression in individuals with the reference genotype. As expected, the distribution of log fold difference in either PSI or expression for individuals carrying the reference allele is centered at 0.
We have clarified this in the methods section and figure legends.

4.It is expectable that most joint seQTLs result from variants directly impacting splicing in cis. As the quantification of splicing is noisier than that of expression, a stronger effect is required for the detection of an sQTL than an eQTL. In other words, joint seQTLs are essentially sQTLs. This illustrated by the example in Figure 4A, with the SNP in an intronic region of the elncRNA being associated with strong differences in splicing and tiny (<1%) and barely significant differences in expression. Moreover, current knowledge and reported evidence strongly suggests that cis regulation of splicing is essentially "local", i.e. directly involves the processed sequences and not the interference of neighbouring RNAs. Similarly, to my knowledge there is no evidence suggesting a trend for genes encoding splicing factors being associated to the same eQTL variants as those of their target RNAs. I would therefore predict that most joint seQTLs result from variants within the elncRNA loci directly impacting their splicing. If this is the case, causal inference analysis will naturally be biased towards more strongly linking the variants with elncRNA splicing and thereby suggesting its causal role. The same rationale applies to scQTLs. The authors need to control for that potential bias in their analyses or explain why there is no bias.

We agree with the reviewer that the quantification of splicing is noisier than that of expression. However, and in contrast with the reviewer's hypothesis, higher "noise" in splicing quantification compared to expression led to weaker associations between splicing and seQTLs, as illustrated in the figure below. This is in line with splicing being measured with higher error rate, which would ultimately lead to smaller detectable sQTL effect than what they would be with perfect measurements. This also demonstrates that since a priori, eQTLs association are stronger, if a bias exists in the causal inference analysis, it should favour detection of non-causal associations. Therefore, our approach is not biased in detecting causal seQTLs.

We agree with the reviewer that this potential bias may be a concern to readers and should be addressed. We have added this analysis to the text (Supplementary Figure S7E) and explained why the causal inference testing approach is not biased in detecting causal seQTLs.

"*To assess whether this approach was biased towards the detection of causal seQTLs we compared the slope and adjusted p-value of the associations between the variant and splicing or expression for all causal seQTLs. As illustrated in Supplementary Figure S7E this analysis revealed there is no evidence that stronger sQTLs would favour causal model predictions.*" (Page 16).

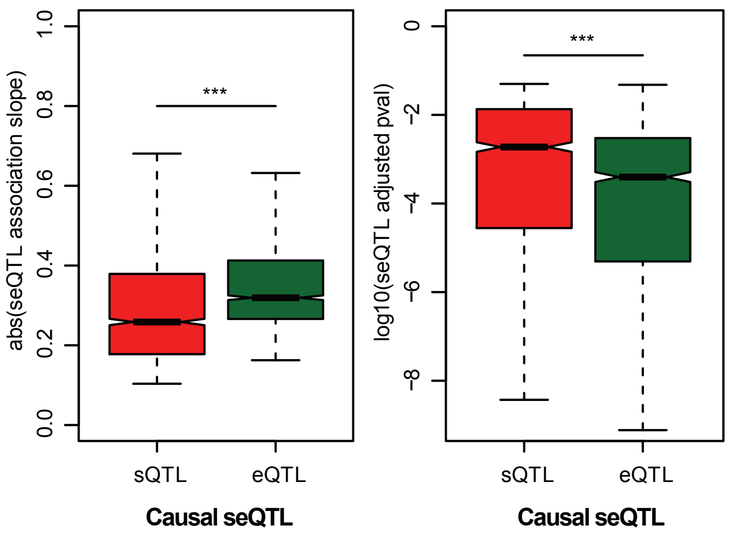

Supplementary Figure S7E. Distribution of the absolute slope (left panel) and the log10 of the adjusted p-value (right panel) of elncRNA sQTL association (red) and target eQTL association (green) for all causal seQTLs.

5.It is not totally clear what message the authors intend to convey with the result of panel 4D. Are they talking about the relative position of the variant to the elncRNA transcript or the target transcript? If the former, shouldn't the known synergy between transcription and 5´ end splicing reflect on elncRNA expression? If the latter, it is not obvious how the result connects to the mentioned synergy.

In Figure 4D, we show the relative position, within a transcript, of the exonic splicing junction which is associated with causal seQTLs. The enrichment in associations with splicing junctions located at 5' end of elncRNAs is consistent with the synergy between 5' end splicing and transcription. We clarify this in the text:

"*Importantly, 90% of seQTL associations that support elncRNA splicing as a mediator of target expression are associated with splicing junctions located at the 5´ end of the transcript, which is consistent with the known synergy between transcription and 5´ end splicing (Furger et al. 2002; Damgaard et al. 2008)(Figure 4D).*" (Page 17).

Proofreading edits:

We would like to thank the reviewer for identifying all the typos listed below. We have corrected them in the revised version of the manuscript.

6.Introduction, page 3, line 13: double "in".

7.Figure S2A, leftmost panel X-axis label: "intrno" instead of "intron".

8.Results, page 10, line 30: remove "of".

9.It is 5´ and 3´(prime) not 5' and 3' (apostrophe).

Other suggestions:

10.Violin plots (with included boxplots) would more comprehensively convey the differences in distributions than the chosen notched boxplots.

We thank the reviewer for this suggestion. Although we appreciate the added information a violin plot can provide, this also renders, in our opinion, their interpretation less intuitive. Because boxplots are simpler and easier to interpret, after consideration, we decided to continue using these to represent the distribution of the data.

Reviewer #1 (Significance (Required)):

It is hard for me to assess the significance of this work (beyond some evidence for the potential functional relevance of the splicing of elncRNAs) until the aforementioned concerns are addressed but it is of potential interest to the broad RNA research community.

I am a computational biologist with experience in the analysis of high-throughput transcriptomic data and a focus on transcriptional and alternative splicing regulation.

==

Reviewer #2 (Evidence, reproducibility and clarity (Required)):

This manuscript addresses an interesting question - whether the splicing of transcriptional enhancer-associated RNAs influences their transcriptional enhancement activity. The analyses appear carefully done, using appropriate datasets and statistical methods. The authors find, for example, that marks of active chromatin are enriched near spliced elncRNAs, that splicing-related motifs of elncRNAs are under selective constraint, and that splicing of elncRNAs is associated with higher elncRNA expression and to very slightly higher expression of target genes.

We thank the reviewer for the constructive feedback on our manuscript. We have extended our analysis to address the reviewers concerns that we detail in our response to the comments below.

However, I did not find the main results convincing of the main conclusion for the following reasons:

1.The most direct evidence is shown in Figure 3, where SNPs that occur in 3' splice sites of elncRNA introns are explored, and it is shown that variants predicted to disrupt splicing of elncRNA introns are associated with reduced expression of target but not non-target genes. But

the fold difference in expression of target genes is extremely small - a few percent - and is actually less than the fold difference in expression of the elncRNAs themselves (which appears closer to 10%), raising the question of whether elncRNA expression rather than splicing may be more important for activity. Furthermore, the entire analysis has an anecdotal quality, being based on only 4 splice-disrupting elncRNA variants. I did not find the figure at all convincing of the conclusion the authors draw from it.

We agree with the reviewer that our analysis is limited by the available genotyping data that is restricted to common genetic variants. Our evolutionary constraint analysis (Figure 2) indicates that variants that disrupt elncRNA splicing are depleted by natural selection and so we expected to identify a relatively small number of elncRNAs (n=4) suitable for this analysis. Despite the anticipated challenges in identifying elncRNA splice site mutations, we nevertheless believe this unbiased natural mutational analysis is analogous to experimentally disrupting splice sites of these 4 elncRNA candidates.

Regarding the strength of impact of splicing on target expression: in the absence of a comparable experiment, we could not anticipate the magnitude of the effect. We acknowledge that previous studies, which sought to completely remove splicing by either deleting all elncRNA introns (Yin et al. 2015) or terminating transcription after its 1st exon (Engreitz et al. 2016), were both associated with significantly stronger impact on elincRNA splicing and target expression than what we report here. The analysis we present here involves single nucleotide polymorphisms and so it is not surprising to have resulted in more moderate impact on overall splicing. Furthermore, whether the differences in the impact on target expression between this and previous analysis is the result of stronger effect of complete removal of splicing or a consequence of the genetic changes introduced remains unclear. The small yet consistent decrease in target expression we observed, even with minimal changes in splicing of an unbiased set of 4 candidates, is in our opinion strong evidence that modulation in elncRNA splicing is sufficient to impact, albeit moderately, target expression.
Importantly, we replicated the impact of decreased splicing on target expression of the 4 elncRNA candidates using 89 samples of Yoruba (YRI) population from the Geuvadis dataset (Supplementary Figure S5). The robustness of the mutational study consistently supports the physiologically relevant effect of elncRNA splicing on cognate enhancer function.

As pointed out by the reviewer, elncRNA SS variants led to stronger impact on the expression of the elncRNAs compared to that of their targets (Figure 3F,H and Supplementary Figure S4), which suggests that target expression regulation is likely a consequence of changes in elncRNA expression as a result of changes in its splicing. This is described as our working model in the discussion section of the manuscript.

2.Figure 4 uses a causal inference approach and involves larger datasets. While causal inference can be a useful tool to identify candidate causal relationships, it does not prove causality, which still requires some sort of experimental perturbation. Thus, I found these results suggestive but still not satisfying to justify, e.g., the title of the paper or claims made in the abstract. As in Figure

3, the specific example shown in Fig. 4A again shows a relatively tiny effect on target gene expression, which again appears to be a few percent at most.

For the reasons explained above, we had no expectation that the effect size of the association between elncRNA splicing and target expression would be high. It is nevertheless key that these associations are robust, which would provide reliable support for our hypothesis. To assess this, we used 2 independent datasets to replicate elncRNA target associations with sQTL variants associated with elncRNA splicing: 1) 147 LCL samples from GTEx and 2) 31,684 blood samples from eQTLgen. Using these datasets, we replicated the association between sQTL and target expression for targets of up to 77% of elncRNAs. As expected, replicated associations have significantly higher effect size in both datasets (Supplementary Figure S9) and 1.2 times more associations can be replicated in the eQTLgen blood samples with a larger cohort size. LCL-specific effect of elncRNA splicing likely explains why not all associations are replicated in these blood samples. We report these analyses in the manuscript (Supplementary Figure S9).

We agree with the reviewer that the causal inference analysis is only suggestive per se. However, we would argue that conclusions of the present manuscript do not rely on this analysis alone, but instead on the combined evidence of several experiments, including the natural mutational analysis that is analogous to the experiment the reviewer proposes.

Considering the reviewers concern, we realized that previous version of Figure 4A did not reflect the average strength of the association between seQTL variant and target expression (median=0.319, ranging from 0.16 to 0.81, Rebuttal Figure 1). For this reason, we replaced the previous illustration by a more representative example (Figure 4A).

The text illustrating reproducibility of our results in GTEx and eQTLgen have been added to Page 17 of the manuscript.
"*We used two independent datasets to assess the robustness of elncRNA target association with sQTL variants we predict to be associated with the splicing of these elncRNAs in LCLs. Using a smaller cohort of LCLs (n=147 (GTEx Consortium 2013)), we found a significant association in the same direction between sQTL and target expression for targets of 70% of elncRNAs (45% of variants). A larger fraction of associations (77% of elncRNAs and 52% of variants) could be replicated in a larger cohort of blood samples (n=31,684 (Võsa et al. 2018)). The difference in size between these two cohorts is likely to explain the difference in replication rate. The association between elncRNA splicing variants and target expression that were replicated have significantly higher effect size relative to non-replicated associations (Supplementary Figure S9). Furthermore, LCL-specific effect also likely explains why not all associations can be replicated in the large blood cohort.*" (Page 17).

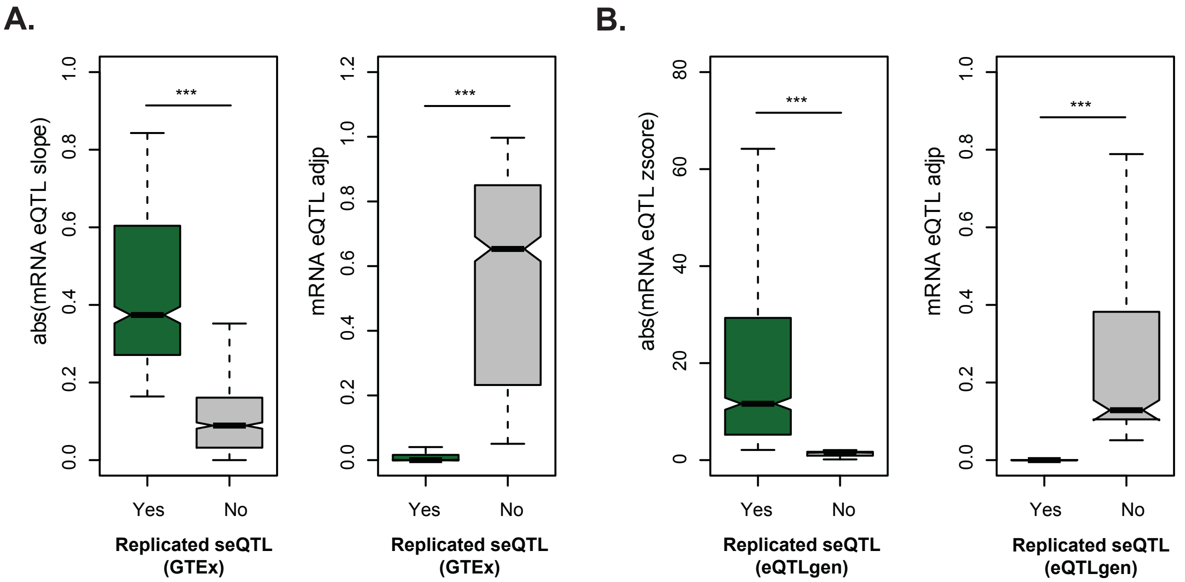

Revised Supplementary Figure S9. Distribution of the absolute slope or z-score (left panels) and adjusted p-value (right panels) of elncRNA target eQTL associations that can be replicated (after multiple-testing correction <5% and in the same association direction, green) or not (grey) using LCL samples from GTEx (A) and blood samples from eQTLgen (B).

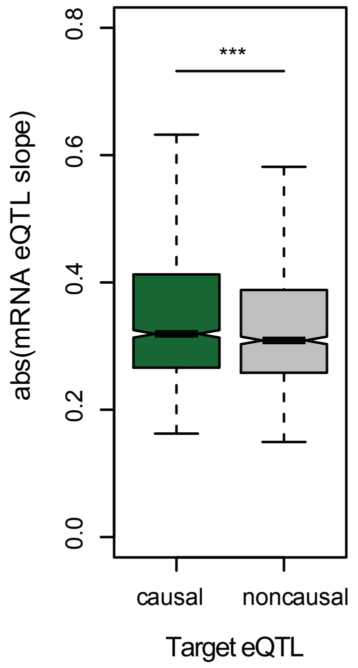

Rebuttal Figure 1. Distribution of the absolute slope of target eQTL associations that are predicted to be causally (green) or non-causally (grey) impacted by elncRNA splicing.

.

A.

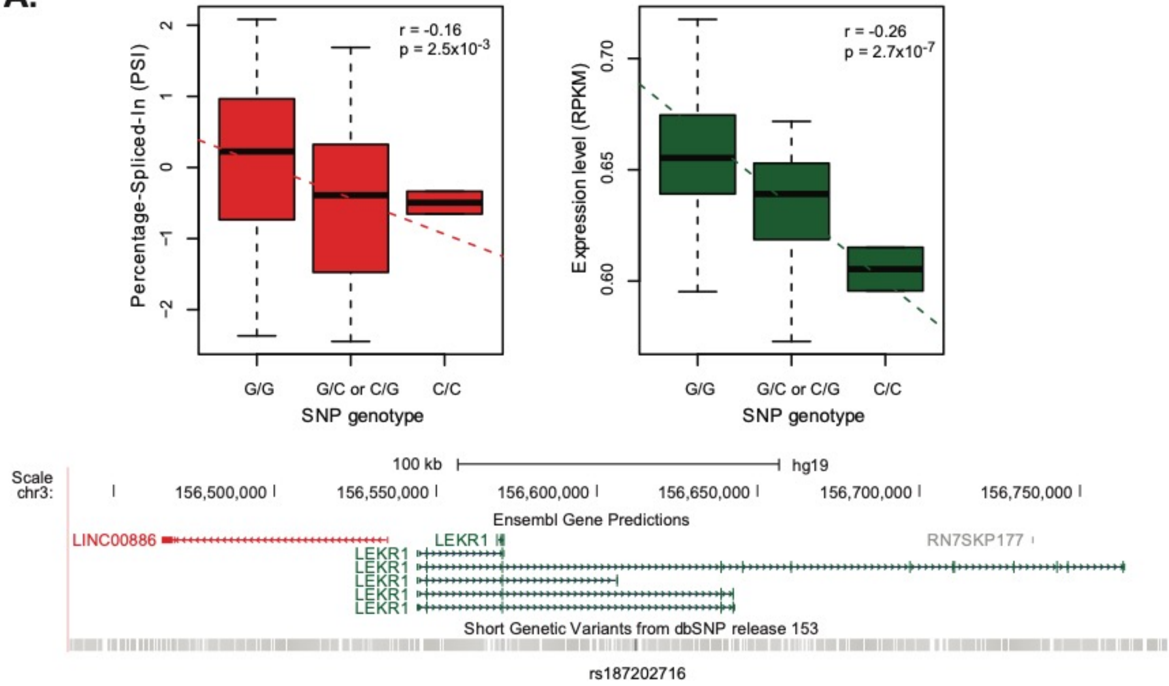

Revised Figure 4A. LINC00886 (ENSG00000240875) is a multi-exonic elncRNA whose splicing is associated with genotype of the SNP variant (rs187202716), which is also associated to the expression level of an elncRNA target, LEKR1 (ENSG00000197980). (Top panel) Distribution of the Percentage-Spliced-In (PSI) of elncRNA splicing (LINC00886, red) and its target expression (LEKR1, green) in samples across the population that carry different alleles of the SNP variant (rs187202716). Spearman's rho and p-values are shown. (Bottom panel) Genome browser illustrating the genomic positions of the elncRNA (LINC00886, red), its target (LEKR1, green), and their associated SNP (rs187202716, black).

3.Figure 2 shows that splicing-related signals are under selective constraint in spliced elncrRNAs, which is convincing. But this does not prove that splicing of elncRNAs is directly related to enhancer activity. It is equally plausible that elncRNA expression directly impacts enhancer activity and that elncRNA splicing is conserved because it boost elncRNA expression, for example.

The reviewer is right and the sentence "If splicing of elncRNAs is important for enhancer function, …" does not faithfully describe the conclusions that can be drawn from the analysis reported in Figure 2. This portion of the text now reads: "If splicing of elncRNAs is functionally relevant, one would expect selection to have prevented the accumulation of deleterious mutations in their splicing-associated motifs during evolution" (Page 8). We would like to thank the reviewer for pointing this out.

Other points:

4.Are the ChiP profiles in Figures 1A-E significantly different from each other in a statistical sense? Probably yes, but a specific test should be done.

We now added boxplots representing the distribution of read density centered at transcript promoters. Statistical difference in the distribution is also tested. We show this in the revised Figure 1A-E and Supplementary Figure S1B-C.

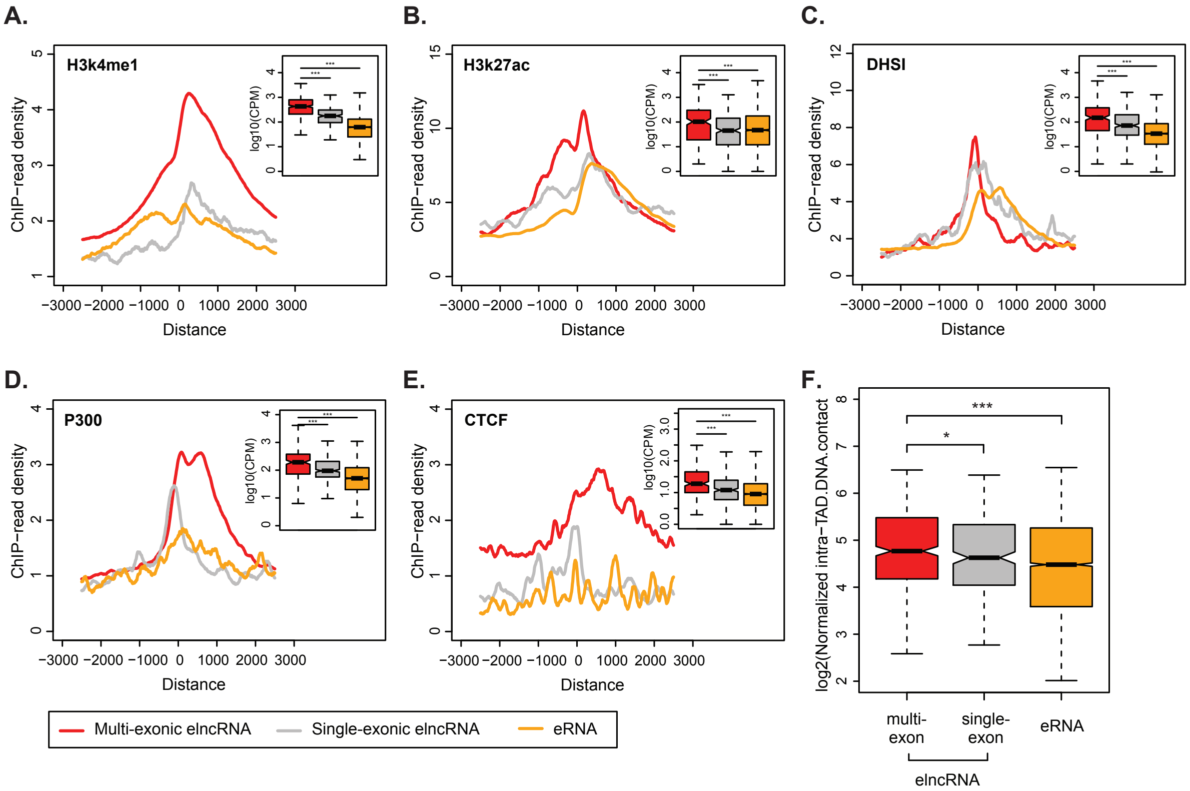

Revised Figure 1. Multi-exonic elncRNAs are transcribed from highly active enhancers. Metagene plots and distribution (figure insets) of (A) H3K4me1, (B) H3K27ac, (C) DNase I hypersensitive sites (DHSI), (D) P300 and (E) CTCF ChIP-seq reads in LCLs at promoters of multi-exonic (red) and single-exonic (grey) elncRNAs, and eRNAs (yellow). (F) Distribution of the average amount of DNA:DNA contacts within LCL TADs containing multi-exonic (red) and single-exonic (grey) elncRNAs and eRNAs (yellow). Differences between groups were tested using a two-tailed Mann-Whitney *U* test. * $p < 0.05$; *** $p < 0.001$.

5.This sentence (p. 10) was hard to follow and should be clarified: "As expected, the impact of SS variants on gene splicing efficiency depends on the total number of alternative transcripts and exons and ranges from 11% to 24% for elncRNA with 6 to 2 number of exons, respectively (Supplementary Figure S3)."

We had previously estimated the average amount of change in splicing for all alternative splicing events at each elncRNA candidate. To calculate this, we considered the difference in Percentage-

Spliced-In (PSI) for all splicing events and divided this by the total number of considered events. Given that only a subset of events is affected by a splice site variant, the more exons an elncRNA has, the more alternative splicing events are likely to occur and the lower the average impact of a SS variant on overall gene splicing efficiency is expected to be. Following a comment from reviewer 1 (comment 1), we now only consider splicing events directly disrupted by the SS variant. We agree this sentence was not clear and have removed it from the manuscript.

6.Related to point 5 above, Supplementary Figure 3 is somewhat confusing because two splicing change and three expression change plots are shown for each locus, without labels of what each one is, or explanation of what the red and green colors mean.

We apologize for the confusion and thank the reviewer for pointing this out. In the figure, we plot the fold difference in elncRNA splicing, target gene splicing, target gene expression, non-target gene expression, and elncRNA expression. elncRNA features are plotted in red and target gene features are plotted in green. We have added labels to clarify the relevant plots (Figure 3D-H, Supplementary Figure S4,5).

Minor points:

1.Top of p. 16: "90% of those that support elncRNA splicing as a mediator 3 of target expression are located at the 5' end of the transcript, which is 4 consistent with the known synergy between transcription and 5' end splicing" - a reference is needed

We thank the reviewer for pointing this out and we have now added the appropriate reference.

"*Importantly, 90% of seQTL associations that support elncRNA splicing as a mediator of target expression are associated with splicing junctions located at the 5´ end of the transcript, which is consistent with the known synergy between transcription and 5´ end splicing (Furger et al. 2002; Damgaard et al. 2008)(Figure 4D).*" (Page 17)

2.Figure 5B,C y-axes indicate "fold difference", but scales include negative numbers, which is confusing. Probably should redo the analysis showing log of fold difference.

We thank the reviewer for the suggestion. Since the fold difference in Percentage-Spliced-In (PSI) used to estimate the amount of splicing at each exon junction can be of both positive and negative values, we now plot the log modulus transformation (John and Draper, 1980) of the data, which is equivalent to a log transformation while preserving the sign of the data. The analysis has been redone for Figure 3D-H, 5B,C, and Supplementary Figure S4, S5. This change does not impact the conclusions and makes the interpretation of the results more intuitive.

3.p. 20 Describes U1 snRNP as "a protein essential for the 4 recognition of nascent RNA 5' splice site and assembly of the spliceosome". U1 is a large RNA-protein complex, not a protein.
We thank the reviewer for pointing this out and this has now been corrected.

"*Chromatin-bound lncRNAs have been recently shown to be enriched in U1 small nuclear ribonucleoprotein (snRNP) RNA-protein complex, a protein essential for the recognition of nascent RNA 5´ splice site and assembly of the spliceosome (Yin et al. 2020).*" (Page 22)

4.Typo: p. 11, l. 2 missing word (genes): "expression levels of other nearby was unaffected"
This has been corrected.

Reviewer #2 (Significance (Required)):

The question addressed is very interesting, given recent work the significance of transcription from enhancers, and work addressing functional relationships between splicing and expression. The work is suggestive of effects of enhancer splicing on expression but I did not find it fully convincing as the effects observed are extremely small, and other explanations are not ruled out, as discussed above.

Prior literature has shown that many active enhancers are transcribed, that enhancer transcription can preced and is positively correlated with target gene expression, and work from both Ulitsky and from the authors indicates that splicing of enhancer-associated lncRNAs is positively correlated with enhancer activity. A variety of studies have also shown that splicing of protein-coding genes generally has a strong positive effect on gene expression. Here, the authors attempt to go further and show that splicing of enhancers causes increased transcriptional enhancement of target genes. A variety of public genotype, expression, chromatin and other types of data are analyzed to address this question. The statistical genetics crowd may find the work of interest, but molecular biologists will not be convinced of the conclusions. My expertise is in computational biology, genomics and RNA biology.

Engreitz JM, Haines JE, Perez EM, Munson G, Chen J, Kane M, McDonel PE, Guttman M, Lander ES. 2016. Local regulation of gene expression by lncRNA promoters, transcription and splicing. *Nature* **539**: 452-455.

John, J., & Draper, N. (1980). An Alternative Family of Transformations. *Journal of the Royal Statistical Society. Series C (Applied Statistics), 29*(2), 190-197. doi:10.2307/2986305

Li YI, Knowles DA, Humphrey J, Barbeira AN, Dickinson SP, Im HK, Pritchard JK. 2018. Annotation-free quantification of RNA splicing using LeafCutter. *Nature genetics* **50**: 151-158.

Yin Y, Yan P, Lu J, Song G, Zhu Y, Li Z, Zhao Y, Shen B, Huang X, Zhu H et al. 2015. Opposing Roles for the lncRNA Haunt and Its Genomic Locus in Regulating HOXA Gene Activation during Embryonic Stem Cell Differentiation. *Cell stem cell* **16**: 504-516.
