# Supplementary material for: The activity of human enhancers is modulated by the splicing of their associated lncRNAs
Source: PLoS Comput Biol. 2022 Jan 11;18(1):e1009722. doi: 10.1371/journal.pcbi.1009722 (PMC8803168; doi:10.1371/journal.pcbi.1009722)
Supplement: Attachment — Submitted filename: Jennifer_Tan_Response_To_Reviewers.docx [file pcbi.1009722.s014.docx]

**We would like to thank the reviewers for their constructive feedback. We respond to all the reviewers points below. We highlight major changes introduced to the manuscript in response to both reviewers’ comments in the attached revised version of the manuscript.**

**Reviewer #1**: I praise and thank the authors for addressing my concerns in a mostly satisfactory way. My remaining requests for revision aim to improve the clarity of the description of the splicing analyses and results, namely by the correction of some misleading statements. In the way the manuscript is currently written, there is an apparent mix-up of concepts related to splicing efficiency and alternative splicing quantification.

We would like to thank the reviewer for considering the manuscript and the suggestions we implemented and that have improved the clarity of the manuscript.

Minor essential revisions:

1. The splicing community commonly uses PSI (percent spliced-in) as a metric for quantification of alternative splicing that is computed based on the profiling of alternative exon-exon (i.e. spliced) junctions. This is, to my knowledge, how LeafCutter operates, using split reads (i.e. reads covering exon-exon junctions) to uncover alternative intron-excision options and the PSI values provided therein and used in Figures 3, S3 and S4 are ratios involving only spliced transcripts. The authors chose to also call PSI to a splicing index (originally named coSI in the Tilgner 2012 paper) that involves a ratio between (split) reads spanning exon-exon junctions and (unsplit) reads covering exon-intron junctions, as a metric for splicing efficiency. Although those two quantifications are not totally independent (the more efficient the splicing, the more exons get included), their conceptual difference is very important for our understanding of splicing efficiency and mechanisms of alternative splicing regulation, which are also different concepts. The two PSI metrics need to be clearly disambiguated.

We have now explained and distinguished the concepts of PSI, as used by LeafCutter, and splicing index, which is now changed to its original name, coSI, as in the Tilgner 2012 paper.

“This increase in density of splicing-related motifs in elncRNAs support their increased splicing efficiency, as estimated using completed splicing index (coSI), which represents the ratio of reads that span exon-exon splice junctions (excised intron) over those that overlap exon-intron junctions (incomplete excision) (Tilgner et al. 2012), relative to plncRNAs (Supplementary Figure S2E, p=5x10^-11^, two-tailed Mann-Whitney *U* test), consistent with what was previously reported in mESCs (Tan et al. 2020).” (Page 7)

“To estimate the impact of SS variant on splicing efficiency, we calculated the Percentage-Spliced-In (PSI) (Li et al. 2018) per individual and for each elncRNA splicing event involving the splice donor or acceptor site disrupted by the SS variants (Figure 2B,C, Supplementary Figure S3). PSI measures exon exclusion by considering spliced reads spanning exon junctions (Li et al. 2018).” (Page 9)

2. Exon “skipping” means exon “exclusion”, not “inclusion”. This would require correction of the newly written lines 25-28 of page 10, as they currently convey contradictory messages, and of whatever other sentence in the manuscript where “skipping” may be misused. However, even after such correction, those two sentences are misleading. The fact that some log-fold differences in PSI for the alternative allele are positive does not mean that the variants can promote more inclusion, as their PSIs are compared to a median reference genotype sample and not the reference genotype in the same individuals. In other words, those individuals with positive log-fold differences could have higher-than-average PSIs if they had a reference genotype and therefore still have a decrease from a much-higher-than-median to a slightly-higher-than-median PSI with the change in genotype. As the comparisons are not “paired” and therefore do not control for the trans-regulation of splicing, statements can only be made about the distributions as a whole (i.e. their average behaviours) and not about individual values.

We have corrected this section that now reads:

“Alongside increased exon exclusion, SS variants can also promote other alternative splicing events (Figure 2B,C, Figure S3). Despite some increase in exon skipping, SS variants are associated with an overall decrease in splicing events (Figure 2D and Supplementary Figure S4).” (Page 9)

Optional/discretionary edits:

3. I disagree with the authors’ statements, not justified in the rebuttal, that violin plots have a “less intuitive” interpretation and boxplots are “simpler and easier to interpret”. It is hard to understand how a plot that conveys a smoothed histogram of the full distribution can be less intuitive, particularly when it can be plotted behind a boxplot to convey quartiles. The choice of less informative plots is even more intriguing in a Computational Biology journal. Having said this, it is an issue that does not affect this manuscript’s suitability for publication. My remark only intends to promote a good practice that would bring extra scrutinisability to the results of this work.

We have changed all boxplots in our figures to violin plots overlayed by boxplots in our figures.

4. Plots involving p-values (i.e. significance) in Supplementary Figure S7 would be more easily interpretable by the readers if -log10 (instead of just log10) was used. Higher significance should show up higher in the plots. I apologize for having overlooked this in the first review.

We have changed plots representing log10 of p-values to -log10 of p-values in Supplementary Figure S7 and S9.

**Reviewer #2:** The authors have revised their manuscript, but the results lack novelty and the data do not compellingly support the conclusions drawn.

We would like to thank the reviewer for considering the revised version of the manuscript. We respectfully disagree with the reviewer’s conclusions that 1) our study lacks novelty and 2) our findings are not supported by compelling evidence.

1) The reviewer refers to earlier studies by us and others to support the conclusion that our work lacks novelty. These studies we cite and the reviewer refers to describe the association between elncRNA splicing and cognate enhancer activity. Here we address an open question in the field: whether this association reflects a causal role of elncRNA splicing in enhancer activity or else if it’s simply a consequence of increased enhancer activity leading to higher transcription and increased levels of splicing noise at these loci. We showed using a genome-wide multi-omics approach, that elncRNA splicing directly impacts cognate enhancer chromatin status and activity.

Our study further illustrates that large cohort genotype and expression data can be leveraged in a novel way to conduct genetics experiment, *in silico*. The novel approach we describe here can be used to address questions pertaining to different mechanism of gene regulation and we trust will inspire the general genomics, population genetics and transcription regulation/RNA communities.

2) The conclusions presented are supported by multiple and compelling lines of evidence. Specifically: We show that elncRNA splicing but not exon sequence is preserved by natural selection (Figure 2); we found that the expression of target genes is decreased in individuals carrying variants that disrupt (Figure 3) or are associated with decreased elncRNA splicing (Figure 4). Supporting the direct role of splicing on enhancer activity, we found decrease in elncRNA splicing is associated with changes in chromatin status at their cognate enhancers (Figure 5). In response to the reviewer’s comments and to investigate the robustness of our findings, we replicated the analysis in multiple independent datasets as is common practice in the field. The results of the independent replication analysis are presented in Supplementary Figures S7 and S9.

Regarding novelty, Figure 1 of the manuscript is virtually identical in content to Figure 2 of a paper recently published by the authors (<https://www.life-science-alliance.org/content/3/4/e202000663>), showing essentially the same result in a slightly different system. So this result is purely confirmatory and should perhaps be a supplementary figure.

We have moved the results of Figure 1 to Supplementary Figure 1 as suggested by the reviewer. We have kept the description of the results in the main text to demonstrate that similar to what was observed previously in a mouse cell line and other human cells, the association between elncRNA splicing and target expression is also detectable in LCLs. Without a measurable association in LCLs, this system would not be a suitable model to address whether elncRNA splicing modulates enhancer activity or else is a consequence of increased accessibility of highly active enhancers.

Figure 2 shows an evolutionary result that supports that splicing of elncRNAs is under purifying selection, but provides no evidence of what the function of this splicing might be, so is novel but plays a minor role in advancing the authors' overall argument.

We agree with the reviewer that evidence that natural selection preventing the accumulation of mutations at elncRNA splice sites and splicing related motifs is novel, but per se is not sufficient to support the direct role of elncRNA splicing on enhancer activity. However, evidence that elncRNA processing being preserved by natural selection supports that this process is not simply the result of increased noisy transcription and splicing at highly active enhancers. This is important to address the central question of our work: whether elincRNA splicing is functionally relevant. We have amended the conclusion of this section to clarify this point.

“Evidence that selection has purged deleterious mutations at elncRNA splicing-associated motifs during mammalian and recent human history, supports the functional relevance of elncRNA processing and argues against it being simply the consequence of increased spurious transcription and splicing at highly active enhancers.” (Page 8)

Thues, the main direct evidence to support the authors' main conclusion that splicing of elncRNAs increases enhancer activity is still in Figure 3. And still the data are extremely weak. The analysis is still based on an n of 4, and the effects are extremely small. Alternate possibilities such as that the elncRNA expression rather than splicing modulates enhancer activity are still not excluded. Overall, I don't feel that the authors have improved the manuscript in a substantive way. The data supporting the main conclusions are still not compelling, and the novelty is compromised by their recent publication, cited above.

Our main conclusions are drawn from the results presented in Figure 2, 3 and 4 (previously Figure 3, 4 and 5). Specifically, using single nucleotide polymorphisms at elncRNA splice sites, we found that disrupting elncRNA splicing significantly impacts target gene expression (Figure 3, previously Figure 4). We agree with the reviewer that the number of loci studied in this section is small (n=4) if one views this as a classical genomics experiment. However, this *in silico* experiment is analogous to experimentally disrupting splice sites of selected elncRNA candidates, where groups of individuals within the population carrying different alleles are natural biological replicates of the experiment. In addition, the relatively number of loci available to carry out this experiment is limited by the depletion of variants that disrupt elncRNA splicing by natural selection, as demonstrated in our evolutionary constraint analysis (Figure 1, previously Figure 2). To gain further support on for our conclusions, we used large genome-wide causal inference testing analysis by investigating the direct impact of elncRNA splicing-associated QTLs (sQTLs) on target gene expression (eQTLs) (Figure 3, previously Figure 4) and on enhancer-associated chromatin signatures (cQTLs) (Figure 4, previously Figure 5). In response to the reviewer’s previous comments and to investigate the robustness of our findings, we replicated the analysis in multiple independent datasets, which fully support our initial conclusions (Figure S7 and S9).

Similar to the reviewer, we also questioned the impact of the well-established link between splicing and increased transcription/transcript abundance on our conclusions. For this reason, we only considered variants associated with elncRNA splicing but not with its expression in the analysis presented in Figure 3 and 4 (previously Figure 4 and 5). In other words, individuals carrying these variants display a significant difference in elncRNA exon inclusion levels, but not elncRNA expression. These analyses allowed us to study the impact induced by elncRNA splicing independent from their expression, and support that the association we observe is a consequence of a role of elncRNA processing.
