# Supplementary material for: The activity of human enhancers is modulated by the splicing of their associated lncRNAs
Source: PLoS Comput Biol. 2022 Jan 11;18(1):e1009722. doi: 10.1371/journal.pcbi.1009722 (PMC8803168; doi:10.1371/journal.pcbi.1009722)
Supplement: Attachment — Submitted filename: Jennifer_Tan_Response_To_Reviewers.docx [file pcbi.1009722.s015.docx]

Reviewer #1: I praise and thank the authors for their efforts in addressing my minor concerns.

Unfortunately, they appear to keep struggling with the concepts and terminology associated with alternative splicing and its quantification. The Percentage-Spliced-In (PSI), as the name indicates, measures exon INCLUSION, and not “exclusion” (that would be 1-PSI or 100%-PSI) as stated in the paper. It is very important that the definitions are accurate and in line with the usual interpretation, so that the readers are not (unintentionally) misled.

We would like to thank the reviewer for pointing this out. We agree this is an important issue and that the definitions should be clear to avoid misleading or confusing the readers.

Our population splicing analysis relies on PSI calculated by LeafCutter (as described in the methods, Li et al. 2018) and we adopted this terminology throughout. LeafCutter PSI measures intron exclusion, as described in their manuscript:

“LeafCutter uses only reads that map across junctions to estimate intron excision rates”;

“LeafCutter finds alternatively excised intron clusters and quantifies intron excision levels in all samples.”

Li et al 2018 Figure 1a

This definition is somewhat different from the original definition of PSI (Barash et al. 2010, Kakaradov et al. 2012) that uses the ratio of RNAseq reads including or excluding exons to measure efficiency of exon inclusion. Intron-centered splicing quantifications as the one implemented by LeafCutter allow one to infer directly from the reads which intron is removed, thereby resolving some of the difficulties that can be found by “standard” exon centered splicing quantifications when splicing events are for example complex (Li et al 2018).

In addressing the reviewers’ comments to the previous version of the manuscript we introduced errors that we have now corrected. To clarify this point, we specify the difference in PSI terminology when we introduce PSI.

We also clarify this point in the revised version of the manuscript and ensure that we are consistent in adopting the terminology used by LeafCutter.

Specifically:

*Page 10: “PSI measures intron excision by considering spliced reads spanning exon-exon junctions (Li et al. 2018). This is different from the original PSI definition (Barrash et al 2010, Kakaradov et al. 2012), which uses the fraction of reads that include or exclude a given exon to measure the efficiency of exon inclusion.”*

Similarly, the confusion between PSI and coSI appears to remain. In Figure 2B,C and Figure S3, the authors assign differences in PSI to S1 and S2, representing single exon-exon junctions. Based on the PSI definition, it is unclear which alternative exons S1 and S2 refer to or why distributions in actual PSI (instead of fold differences) are not compared. The meaning of ratios between exonic and intronic sequences (indeed putatively associated with splicing efficiency) appear to be confused with ratios between alternative exonic sequences (standard alternative splicing quantifications).

Figures 2B, C and S3 represent differences between alternative intron excision events between individuals carrying the reference or alternative allele at the splice site, as per the PSI definition by LeafCutter. Top panels of the figures that illustrate the splicing events were generated using the R Shiny framework LeafViz, which was developed to visualize LeafCutter differential splicing events (Li et a. 2018). We added this to the methods section.

*Page 25: “Intron-centric splicing efficiency as measured by LeafCutter is visualized by LeafViz (Li et al. 2018).”*

Along the same lines, what “other alternative splicing events” (page 10, lines 20-21), other than exon skipping, can SS variants promote that a reader can see in Figures 2B,C and S3? What does an “overall decrease in splicing events” mean? Does it mean that that more exons are skipped or more introns are retained?

A decrease in splicing event (specifically an intron excision event) in individuals that carry disrupted splice sites indicates a reduced number of reads, therefore supports the excision of the intron in question and suggests a reduction in splicing efficiency. After careful revision, we agree with the reviewer that this portion of the text was hard to follow and so we have clarified it. In summary, we added this comment to address the observation that decreased splicing at a given splice site due to the presence of alternative SS (intron S1 in Figure 2B) can result in increased splicing at neighbouring sites (intron S2. In Figure B) We found the increased alternative splicing (as seen in neighbouring intron S2 exclusion in Figure 2B) is not significantly higher not sufficient to account for the decrease splicing at S1(Figure 2B,C and Figure S4 (previously Figure S3).This support that SS variants are associated with an overall decrease in splicing efficiency.

*Page 10: “We compared the average difference in PSI, as a proxy for change in splicing efficiency, of all affected splicing events between individuals that carry the reference and alternative canonical splice donor/acceptor sites (GT-AG). We found that SS variants are associated with a significant decrease in splicing events involving polymorphic splice donor/acceptor sites (Figure 2B-D and Supplementary Figure S3). As expected, alongside decreased excision of the affected introns, SS variants are also associated with increased alternative splicing events that involve neighbouring intact splice donor or acceptor sites (Figure 2B,C, Supplementary Figure S4). However, compared to decreased intron excision events observed in individuals carrying the alternative SS variants, the increase in overall alternative splicing events, in the same individuals, is 2-8 times smaller and not statistically significant (Figure 2B,C, Supplementary Figure S4). This supports that the presence of SS variants are associated with an overall decrease in splicing.”*

I strongly recommend that the authors do not underestimate the importance of this issue and thoroughly revise these definitions and quantifications with help from an alternative splicing expert. Until this is done properly, the results from this work, even if sound, will remain hardly intelligible for most readers and, in my view, not fully ready for publication.

We would like to thank the reviewer for the attention given to the revised version of the manuscript and the insightful comments.

Reviewer #4: I appreciate the authors' efforts to address the issues of novelty and evidence for their conclusions, as well as other points raised. This new version of the manuscript is significantly stronger. However, there are a few specific points that should be developed further before publication.

1. Direct evidence of causality. The main evidence supporting the authors’ conclusion that splicing of elncRNAs increases enhancer activity is still aggregated in a very small number of loci (n=4). I agree with the authors that this in silico experiment is “analogous” to experimentally disrupting splice sites of selected elncRNA candidates. However, I do not believe both approaches are equivalent. At this point, I suggest the authors actually test their hypothesis in an experimental setting. They could pick a few candidates to perturbate splice sites elncRNA with punctual mutations that create weaker or stronger splice sites and evaluate the consequences on enhancer’s activity and chromatin structure.

We appreciate that experimental evidence by which creating weaker or stronger elncRNA splice sites impacts enhancer activity and chromatin structure would further support our conclusions. However, these experiments would be hard to set up in our case.

LCLs were chosen for this analysis because of the large extent of population genomics and transcriptomics data available for this cell line. This allowed us to analyse a phenomenon that was already demonstrated experimentally for a small number of elncRNAs (for example by Yin et al, 2015 or Engreitz et al, 2016) in a genome wide and unbiased manner. Because of the richness of the datasets available for these cells, we were also able to further replicate these findings using multiple independent datasets (Supplementary Figure S5, S7). The results of these replication studies fully support our initial conclusions.

In contrast to the rich source of LCL data available for in silico analysis, as illustrated by the extent of results presented in the manuscript, LCLs are not suited for the experimental validation as proposed by the reviewer because they are generally difficult to edit genetically due to low transfection efficiency and slow proliferation rates. Repeating this work in another cell type would not be possible either, because the unavailability of large genomic datasets will make the identification of highly cell type-specific candidate elncRNAs challenging. In addition, our limited access to resources and expertise would restrict our ability to carry out these experiments.

We believe that the computational analysis presented here, which extends previous experimental (for example by Yin et al, 2015 and Engreitz et al, 2016) and computational analysis (Gil and Ulistky 2018 and Tan et al 2019), is sufficient to support the conclusion that efficient elncRNA splicing directly contributes to enhancer activity genome-wide and that this is not simply a byproduct of increased transcription at highly active enhancers. Further work is now needed to determine the precise molecular mechanisms and potential differences between subsets of elncRNAs.

2. Link between splicing and increased transcription. The authors mentioned that they only considered variants associated with elncRNA splicing but not with its expression in Figs. 3 and 4, but do not show the data (sorry in case I missed it). I think that showing that the total elncRNA expression does not change is as crucial as showing that the splicing of the elncRNA does change.

The reviewer is right in that we have not provided the data supporting that the exclusion of sQTLs associated with elncRNA expression (as described in the methods section p25) is sufficient to account for a potential impact of the variant on elncRNA expression.

We have now added the figure showing that sQTLs considered in our analysis are not associated with elncRNA expression (Supplementary Figure S7A).

*Page 12: “To assess the impact of elncRNA splicing on enhancer activity independent of its transcription, we excluded sQTL variants that are also associated with elncRNA expression (eQTLs) from this analysis (Methods) (Supplementary Figure S7A).”*

*Page 25: “To infer the causal relationship between elncRNA splicing and putative target gene expression, we focused on QTLs that are associated with both splicing of elncRNAs (sQTL) and their putative target gene abundance (eQTL), and we refer to these variants as joint seQTLs. elncRNA sQTL variants that were also associated with elncRNA expression level or splicing of their putative cis-target genes were excluded from the analysis (n=21,650 out of 26,741) (Supplementary Figure S7A).”*

Supplementary Figure S7A.

(A) Distribution of the absolute slope (left panel) and the -log10 of the adjusted p-value (right panel) of elncRNA sQTLs that are excluded (grey) or included (green) in the analysis due to their significant associations with elncRNA expression, respectively.
