# Supplementary material for: The activity of human enhancers is modulated by the splicing of their associated lncRNAs
Source: PLoS Comput Biol. 2022 Jan 11;18(1):e1009722. doi: 10.1371/journal.pcbi.1009722 (PMC8803168; doi:10.1371/journal.pcbi.1009722)
Supplement: Attachment — Submitted filename: Jennifer_Tan_Response_To_Reviewers.docx [file pcbi.1009722.s016.docx]

Reviewer #1: I praise and thank the authors for carefully addressing my concerns.

We would like to thank the reviewer for all the previous comments and we are pleased that all concerns from the reviewer has been addressed in our revision.

Reviewer #4: The authors have done a great job addressing my comment on the link between splicing and increased transcription. I appreciate the new Supplementary figure which is quite strong. In addition, I of course understand that disrupting splice sites of selected elncRNA candidates would be a hard experiment to set up and might not be possible in LCL cells. However, I still believe that the evidence in this manuscript to support the main conclusion of causality (which is the take home message and differentiates this paper from previous literature) is not conclusive. I believe that experimental evidence is necessary to support the conclusion that splicing of elncRNAs increases enhancer activity and several labs around the world would be happy to collaborate in such efforts. If the paper should be published without experimental data, I suggest to tone down the conclusions and acknowledge this issue.

We thank the reviewer and are pleased to hear that our revision has adequately addressed the reviewer’s comments. We also appreciate that the reviewer acknowledges the challenges in experimentally testing elncRNA splicing. We have therefore taken on the reviewer’s suggestion to tone down the conclusions. We have also added a sentence to clarify that by using a data-driven approach, the current analysis is hypothesis-generating.

Please see below detailed changes introduced to the revised manuscript to address this concern.

- We provide evidence that efficient and conserved processing of enhancer-associated elncRNAs contributes to enhancer activity. (Page 2)
- We show that natural selection has acted, at the species and population level, to preserve DNA elements required for frequent and efficient elncRNA splicing Importantly, using a genome-wide and unbiased statistical population genomics approach, we demonstrate that elncRNA splicing is associated with cognate enhancer function, contributing to chromatin status and enhancer activity. Our results provides strong evidence that efficient elncRNA splicing contributes to enhancer activity genome-wide. (Page 3)
- Furthermore, nucleotide variants that disrupt elncRNA splicing is correlated with significant reduction in cognate enhancer activity, supporting the functional importance of elncRNA splicing. (Page 6)
- Causal inference support elncRNA splicing mediates target expression. (Page 11)
- We provide evidence that splicing a sizable fraction of enhancer-associate transcripts likely to directly impact the activity of their cognate enhancer activity.
- We anticipate that the use of larger cohorts should result in an increase in the percentage of elncRNAs for which splicing can be more confidently shown to mediate target expression in *cis*. (Page 18)
- The present work provides clues to what might be the molecular mechanism(s) underlying the link between elncRNA splicing and enhancer function. Further experimental work is now needed to dissect the molecular details underlying the role of elncRNA splicing in modulating enhancer activity and investigate the differences between potentially distinct elncRNA classes, for example, by evaluating the consequences on enhancer factor recruitment, chromatin structure and target activity of experimentally perturbing splicing of candidate elncRNA. (Page 19)
